# T-*pattern* analysis of offensive and defensive actions of youth football goalkeepers

**DOI:** 10.3389/fpsyg.2022.957858

**Published:** 2022-09-02

**Authors:** Fernando Santos, João Santos, Mário Espada, Cátia Ferreira, Paulo Sousa, Valter Pinheiro

**Affiliations:** ^1^Instituto Politécnico de Setúbal, (CIEF – ESE/IPS, CDP2T, ESTSetúbal/IPS), Setúbal, Portugal; ^2^Faculty of Human Kinetics, University of Lisbon, Cruz Quebrada, Portugal; ^3^Life Quality Research Centre (CIEQV-Leiria), Rio Maior, Portugal; ^4^Higher Institute of Educational Sciences, Odivelas, Portugal; ^5^Training Optimization and Sports Performance Research Group (GOERD), Sport Science Faculty of Cáceres, University of Extremadura, Cáceres, Spain

**Keywords:** goalkeeper, match, soccer (football), analysis, T-*pattern*

## Abstract

Nowadays, football goalkeepers (GKs) play an important role in the team's organization, namely, considering the offensive and defensive processes. The purpose of our investigation focuses on the notational and T-*pattern* analysis of the offensive and defensive actions of elite young football GKs. The participating GKs (*n* = 3, mean age of 16.6 years) presented 8 years of experience in the specific position, were internationally selected for the national team of Portugal, and competed in the national U-17 championship of Portugal. Thirty football matches were observed. The observational sample consisted of defensive actions (*n* = 225) and offensive actions (*n* = 296). Two observational instruments were used to codify the actions: the observation system of defensive technical-tactical actions of GKs and the observation system of offensive technical-tactical actions of GKs. Both instruments underwent a validation process, and inter- and intra-observer reliability was tested. The codification of the actions was performed with the *LINCE* program, and later the data were exported to Microsoft Excel and *THEME 5.0*. The notational data were analyzed in *SPSS*, and T-*pattern* detection analysis was performed in *THEME 5.0*. The predominant actions of young observed GKs were fundamentally goal defense and participation in the team's offensive process construction through actions performed with the foot and hand. The analysis of T-*patterns* allowed to identify T-*pattern* actions in the defensive actions of goal defense and exit of the goal, as well as related to the defensive set pieces. In the offensive process, the analysis of T-*patterns* reinforced the participation of the GK in the team's first phase of construction and in the execution of goal kicks and actions that start as a result of the actions of the opponent. The GK's defensive actions are mostly focused on the objective of goal defense and offensive actions with the hands and feet are important for their participation in positional attack construction. Our study contributes to a better knowledge of the GK's actions in the competition and is relevant to be considered by the specific position coaches in the training process organization.

## Introduction

Football is currently one of the most popular sports in the world, resulting in an increase in the number of season competitions and matches (Aurélio et al., 2016; Santos et al., 2021a). It requires an efficient collective organization and, simultaneously, specific development of each player with the consideration of individual and group perspectives (Espada et al., 2018). A detailed evaluation of players' performance is of particular interest because the available time and conditions for training sessions are not always desirable for coaches, who always look for strategies to develop athletes' physical and tactical skills and improve their performance in the competitive environment (Santos et al., 2021b).

In modern football, a goalkeeper (GK) has a massive relevance not only in the goal defense, but also in the offensive process participation of the team (Goméz-Millán and Esquiva, 2017). This evidence is related to the evolution of the characteristics of the players in this specific pitch position and to the regulatory changes introduced by the Federation Internationale de Football Association (FIFA). Consequently, the effective execution of technical-tactical actions is crucial in their performance, requiring the improvement of technical skills and the development of the ability to execute them according to the tactical context of the game (López-Gajardo et al., 2020).

It is also essential to work on the specific individual needs of players according to their pitch positions during the football training process to promote the achievement of the necessary required fitness levels to efficiently perform on match days (Espada et al., 2020). Notably, performance analysis is key to collecting information about players and teams, helping the coach make decisions about training and competition (O'Donoghue, 2006) related to GKs, which is essential not only for training to meet their needs for evolution, but also regarding the requirements related to the team's play style (West, 2018).

In the field of research, studies have been carried out to understand the defensive and offensive actions of the GK and to verify how they are influenced considering the level of the opponents (Liu et al., 2015; Sainz de Baranda et al., 2019), the competitive level (López-Gajardo et al., 2020), the game result (Kubayi, 2020), and the status of home or away game (López-Gajardo et al., 2020). Although Ortega and García-Angulo (2015) suggested that the main studied disciplines are motor control, namely, focusing on taking penalties (Furley et al., 2017) and sports medicine, most articles analyze injuries based on players' positions on the pitch (Hägglund et al., 2013) or physiological profiles (Ziv and Lidor, 2011). This is somewhat surprising, considering the GK is the most specialized position in a football team (Frick, 2007), and their actions are considered to have a significant bearing on final match outcomes (Liu et al., 2015).

Nonetheless, in performance analysis, it is extremely important to observe and analyze (Martin et al., 2018) in order to collect key information for the development of players and teams through the training process, preparing them for the requirements of a competition (Sampaio and Macas, 2012), based on the training sessions designed to address the needs of the players and teams, the task where sports coaches play a key role (Rodrigues et al., 2021). Recently, López-Gajardo et al. (2020) verified that in GKs, the defensive fitting action is the one that has more occurrences in the different levels of competition analyzed, being a technique referred to as relevant, since it avoids second finalizations of the opponents and allows ball possession. Regarding the result of the game, Kubayi (2020) verified more occurrences of goal defense actions in the GK to win the game, and other forms of defensive interventions were previously observed with great frequency, namely, the exit of the goal to the crossing (Soares et al., 2018), block (López-Gajardo et al., 2020), and grab the ball (Szwarc et al., 2010). In defensive terms, it was previously observed that the intervention zones of GKs are fundamentally located within the penalty area (Sainz de Baranda et al., 2008; Lapresa Ajamil et al., 2018).

The GK's action with their feet, after recovery of the ball, is essential to change the center of the game to the areas of the lower pressure of the opponent (Barreira et al., 2014). Recently, Sainz de Baranda et al. (2019) observed that in high-level teams, the game with the GK's feet had a great relevance, and Serrano et al. (2019) reported that in La Liga, the prevalence of GK's passes increased throughout the editions of the competition. Among GKs aged 14–16 years, great effectiveness of passing was demonstrated, and there were behavior patterns in these actions with the foot and hands (Lapresa Ajamil et al., 2018). In the offensive process, Santos et al. (2022) observed that the participation of a GK resulted from late passes of teammates, showing fundamental technical actions with the feet in the offensive construction of the team, preferably to the side flanks.

Despite the fact that GKs are nowadays observed as key players who can win or lose football games based on their individual and collective actions, associated to the fact that some of the highest financial transfers in modern football are associated with elite players in this specific football game position, the study of the specific actions of GKs is scarce compared to many other areas of the football game. It becomes important and relevant to analyze the actions associated with the role of GKs in the football game, with a perspective of constituting an important assessment tool for athletes who play in this specific pitch position in the football game and for the coaches in order to create the best training tasks to optimize the individual and collective performance of the team.

Many of the previous developed studies focused on observing the technical and tactical actions of elite senior GK, and research carried out in this specific football playing position focused on top-level GKs (Oberstone, 2010) with scarce scientific output investigating youth football GKs (Sainz de Baranda et al., 2005). This is of relevant interest for the coaching staff aiming for knowledge and the improvement of daily training tasks. Even though some previous studies focused on the notational analysis, the analysis through T-*patterns* provides information related to time and sequential structures of defensive and offensive technical actions. Hence, the aim of the present study was to detect T-*patterns* of offensive and defensive actions of youth football GKs. The following hypotheses were established in our study: (1) in the defensive process, the GK performs more T-*patterns* related to goal defense actions within the penalty area, and (2) in the offensive process, GK performs more T-*patterns* related to actions with the feet in the direction of the side zones of the flanks.

## Methodology

### Observational design

The procedures related to the observational methodology were considered, with the objective of analyzing the defensive and offensive GK actions, and two observational instruments were constructed to codify perceptive behaviors, in an official game situation (Anguera et al., 2018a,b; Chacón-Moscoso et al., 2018), giving the investigation an ecological validity (Portell et al., 2015). The observational design of the study is part of quadrant III, being considered ideographic (participants in GK), follow-up (observations made over the competitive season), and multidimensional (actions categorized into various response levels) (I/S/M) (Anguera et al., 2018a).

The investigation considered the procedures enshrined in the Declaration of Helsinki (Harriss and Atkinson, 2011). The observed GKs were previously clarified about the objectives of the study, and they agreed to participate, while at the same time the legal representatives signed informed consent. The study was approved by the Ethics Committee of the Polytechnic Institute of Leiria (CE/ IPLEIRIA/22/2021).

### Participants

Three U-17 GKs (*M* = 16.6 years of age) participated in the study, competing in the national championship of Portugal. The GKs represented a 5-star club (top-level certification by the Portuguese Football Federation) and were regularly present in the training sessions at the national and international levels. They had 8 years of experience playing in the position and had international participation in international tournaments in the representation of the National Team of Portugal.

The 30 football matches of the entire official football season were observed (GK A-10, GK B-8, and GK C-12), constituting an observational sample of 225 defensive actions and 296 offensive actions. For each GK, defensive actions (GK A-63−28%; GK B-45−20%; and GK C-97−43.11%) and offensive actions (GK A-109−36.82%; GK B-64−21.62%; and GK C-123−41.55%) were analyzed.

### Observational instruments

Two observation systems were used to codify the actions: the observational system of defensive technical-tactical actions and the observational system of offensive technical-tactical actions. Both observation systems underwent a validation process (Santos et al., 2022). Table 1 shows the observational system of defensive technical-tactical actions of GKs, consisting of four criteria and 34 categories.

**Table 1:** Observational system of defensive technical-tactical actions.

**Criterion**	**Category**
Intervention form	Crossing
	Goal defense
	Set pieces
	Goal exit
Technical action	1×1 shot
	1×1 divided
	Frontal attack
	Action as last defense
	High lateral drop deviation
	Deviation lateral creep
	Block
	Deviation to punch
	High deviation
	Enchase
	High reception
	High lateral fall reception
	Reception lateral fall creeping
	Creeping interception
Forms of execution the technical action	1 hand
	2 hands
	Feet
	Chest
	Fists
	1 hand
End action field zones	Field zones 1–10

**Figure 1:**
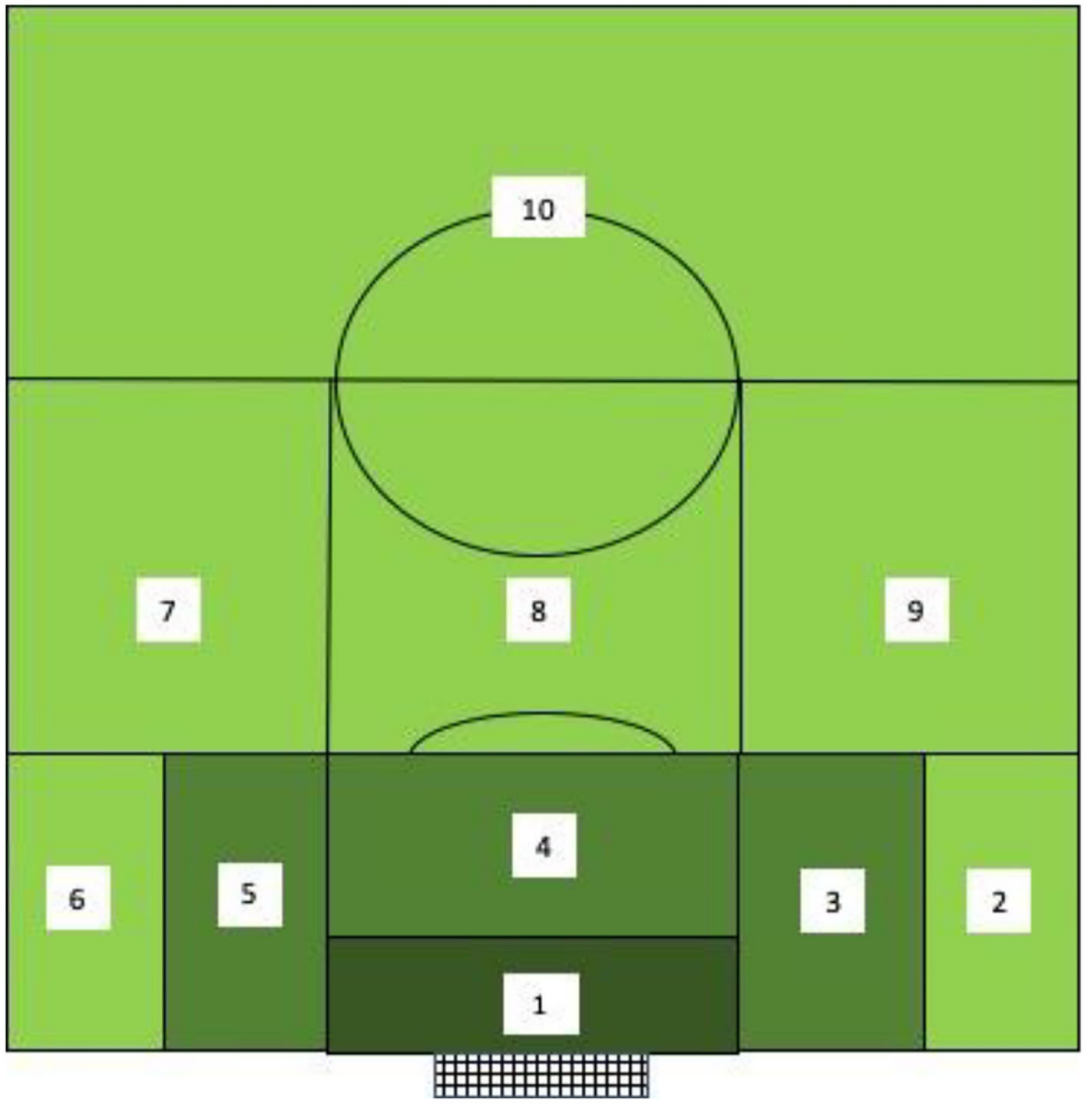
Campogram for defensive technical-tactical actions (adapted from Lapresa Ajamil et al., 2018).

Figure 1 shows us the campogram with the final zones of defensive actions.

The system of observation of offensive technical-tactical actions of GK (Table 2) consists of six criteria and 50 categories.

**Table 2:** Observational system of offensive technical-tactical actions.

**Criterion**	**Categories**
How the ball arrives the GK	Delay
	Opponent's action
	Rules
Zone field start action	Field zones-−1–12
Technical action	Short pass with 2 touches
	Short hand replacement
	Long hand replacement
	Goalkeeper kick
	Long pass at 1st touch
	Long pass at 2nd touches
	Long goal kick
	Short goal kick
	Short pass at 1st touch
	Ball conducting + Short pass
	Ball conducting + Long pass
	Short free kick
	Long free kick
	Short pass with 2 touches
	Short hand replacement
	Long hand replacement
	Goalkeeper kick
Tactical decision	Positional attack
	Fast attack
	Counterattack
End of technical action	Intercepted ball
	Maintenance of ball possession
	Ball out
End field action zones	Field zones 1–12

**Figure 2:**
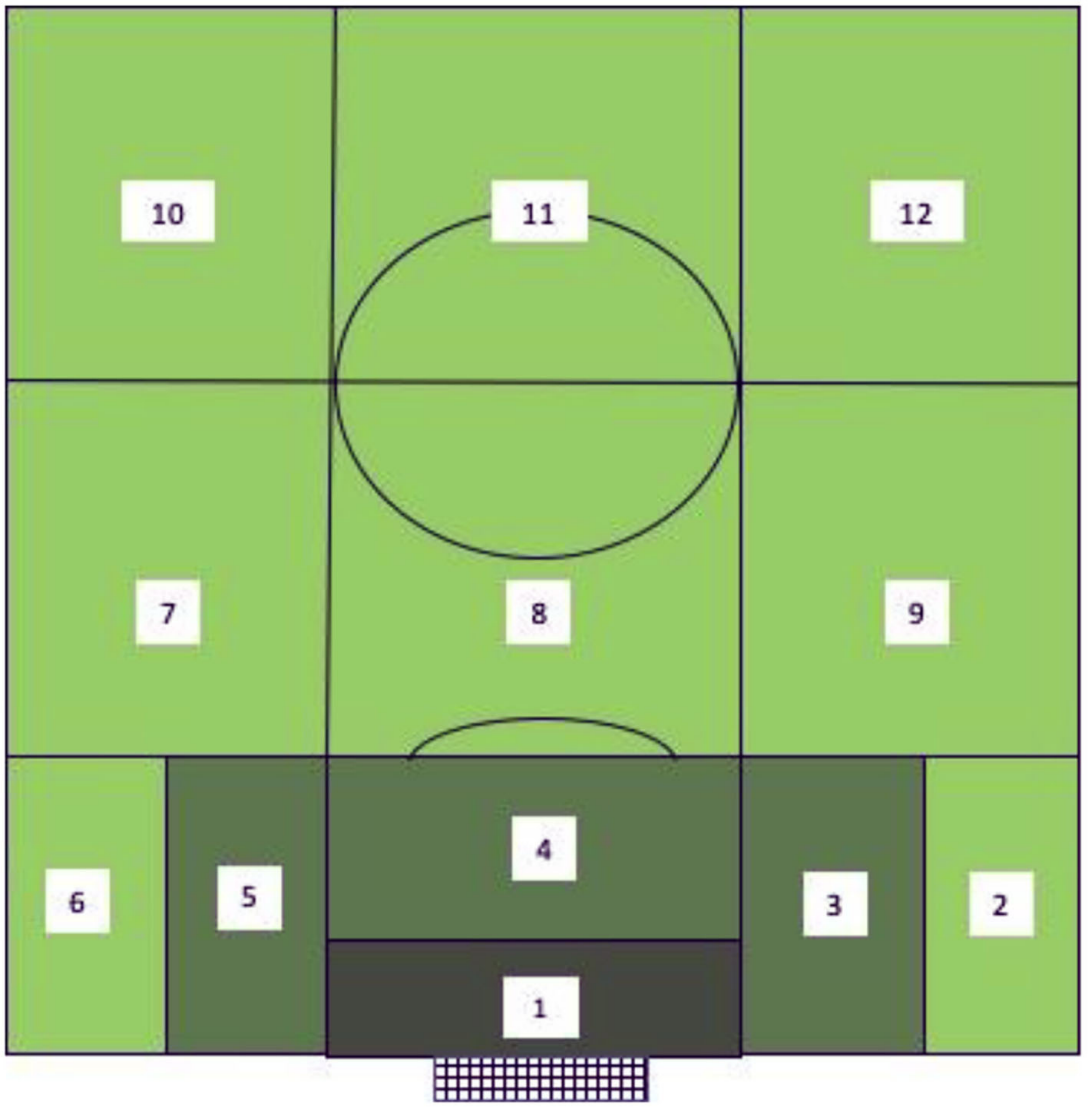
Campogram for offensive technical-tactical actions (adapted from Lapresa Ajamil et al., 2018).

The campogram visualized in Figure 2 demonstrates the start and end zones of offensive actions.

### Reliability

Reliability analysis is fundamental in observational methodology with the objective of ensuring data quality (Blanco-Villaseñor et al., 2014). In the first phase, the observers were trained, followed by the intra- and inter-observer reliability test (Brewer and Jones, 2002). Reliability testing was performed using Cohen's K agreement measure (Cohen, 1960), using the LINCE program (Hernández Mendo et al., 2014). Intra- and inter-observer reliability values above 0.80 were verified (Table 3) in all criteria, and on average, the values were found to be above 0.88.

**Table 3:** Intra- and inter-observer reliability.

		**Inter-observer**	**Intra-observer 1**	**Intra-observer 2**
**Observation system**	**Criterion**	***K***	***K***	***K***
Observational system of offensive	Intervention form	0.87	1.00	1.00
technical-tactical actions	Technical action	0.88	0.90	0.81
	Forms of execution the technical action	0.90	0.82	0.82
	End action field zones	0.88	0.90	0.90
	*Mean*	0.88	0.91	0.88
Observational system of offensive	How the ball arrives the GK	1.00	0.96	1.00
technical-tactical actions	Zone field start action	0.95	0.84	0.93
	Technical action	1.00	0.88	0.97
	Tactical decision	0.82	0.84	1.00
	End of technical action	1.00	1.00	0.90
	End field action zones	0.95	0.86	0.95
	*Mean*	0.95	0.90	0.96

### Procedures

The collected images, for later coding, were retrieved through the camera (Sony HD—HDCR—CX240 9.2 megapixels), placed on a tripod, and positioned in a high plane with an open angle, so that the beginning and end of each action could be identified. The images after being collected and edited were encoded using the *LINCE*^®^ computer program (Gabin et al., 2012). Subsequently, the data were exported from *LINCE*^®^ to the *THEME 5.0*^®^ program to make the T-*pattern* analysis.

### Data analysis

The defensive and offensive action patterns of GKs were analyzed using the *THEME 5.0*^®^ program. The detection and analysis of T-*patterns* enable the identification of the sequence of events that repeat in the same order and in a relatively invariable time distance. In this sense, the concept of the critical interval is essential for the design of time structures and sequences of a series of data. The algorithm for building and identification T-*patterns* is based on critical interval detection, patterns building, and complete patterns competition. This algorithm works from the bottom up, level by level, by eliminating partial and equivalent T-*patterns* (Magnusson, 2000, 2020; Casarrubea et al., 2015, 2018). The criteria defined for the selection of detected T-*patterns* were as follows: (a) significance level 0.005 (*p* < 0.005); (b) the number of occurrences of T-*pattern* ≥ 3; (c) redundancy reduction setting at 90%; (d) deactivation of free heuristic critical interval setting; (e) T-*pattern* validation through data randomization on five occasions, proceeding to compare the randomization data with the real data; and (f) randomization through the simulation filter according to the defined significance level.

## Results

The results presented are related to the T-*patterns* of defensive and defensive actions. Tables 4, 5 show all the recorded T-*patterns*.

**Table 4:** T-*patterns* related to the GK defensive actions.

**T-*pattern***	**Occurrences (** ***n*** **)**			
	**GK A**	**GK B**	**GK C**	**Total**
Goal defense—High lateral drop deviation-−2 hands—End zone 1	6	3	7	**16**
Goal defense—High lateral drop deviation-−1 hand—End zone 1	-	-	3	3
Goal defense—High lateral drop deviations-−2 hands—End zone 4	-	-	3	3
Goal defense—High deviations-−2 hands—End zone 1	-	-	3	3
Set pieces—High deviation-−2 hands—End zone 4	-	5	8	**13**
Set pieces—High deviation-−2 hands—End zone 1	-	-	8	8
Set pieces—High reception-−2 hands—End zone 4	4	-	4	8
Set pieces—High reception-−2 hands—End zone 1	-	-	4	4
Crossing—High reception-−2 hands—End zone 4	-	5	-	5
Crossing—High reception-−2 hands—End zone 1	-	-	4	4
Crossing—Enchase—Chest—End zone 4	-	-	4	4
Goal exit-−1×1 shot-−2 hands—End zone 5	-	-	4	4
Goal exit-−1×1 divided—Chest—End zone 4	-	-	4	4
Goal exit—Frontal attack-−2 hands—End zone 4	-	5	9	**14**

The bold values indicate the T-patterns with the most occurrences.

**Table 5:** T-*patterns* related to the GK offensive actions.

**T-*pattern***	**Occurrences (** ***n*** **)**			
	**GK A**	**GK B**	**GK C**	**Total**
Rules—Start zone 1—Long goal kick—Positional attack—Maintenance of ball possession—End zone 10	8	-	6	**14**
Rules—Start zone 1—Long goal kick—Positional attack—Maintenance of ball possession—End zone 12	7	-	4	**11**
Rules—Start zone 1—Long goal kick—Positional attack—Intercepted ball—End zone 12	-	-	5	5
Rules—Start zone 1—Long goal kick—Positional attack—Intercepted ball—End zone 11	-	-	3	3
Delay—Start zone 8—Ball conducting + Long pass—Positional attack—Maintenance of ball possession—End zone 10	-	-	4	4
Delay—Start zone 8—Long Pass at 1st touch—Positional Attack—Maintenance of ball possession—End zone 10	-	-	4	4
Delay—Start zone 3—Short pass with 2 touches—Positional attack—Maintenance of ball possession—End zone 8	3	-	-	3
Delay—Start zone 3—Short pass with 2 touches—Positional attack—Maintenance of ball possession—End zone 9	3	-	-	3
Delay—Start zone 7—Long pass at 2nd touch—Positional attack—Maintenance of ball possession—End zone 10	3	-	-	3
Delay—End zone 3—Short pass 2 touches—Positional attack—Maintenance of ball possession—End zone 10	3	-	-	3
Delay—Start zone 7—Short pass with 2 touches—Positional attack—Maintenance of ball possession—End zone 8	-	3	-	3
Opponent's action—Start zone 4—Short hand replacement—Positional attack—Maintenance of ball possession—End zone 8	7	-	-	**7**
Opponent's action—Start zone 4—Short hand replacement—Positional attack—Maintenance of ball possession—End zone 7	4	-	-	4
Opponent's action—Start zone 4—Short hand replacement—Positional attack—Maintenance of ball possession—End zone 5	4	-	-	4
Opponent's action—Start zone 4—Short pass with 2 touches—Positional attack—Maintenance of ball possession—End zone 8	-	3	-	3
Opponent's action—Start zone 4—Goalkeeper kick—Counterattack—Intercepted ball—End zone 11	-	3	-	3
Opponent's action—Start zone 4—Short pass with 2 touches—Positional attack—Maintenance of ball possession—End zone 7	-	3	-	3

The bold values indicate the T-patterns with the most occurrences.

It is possible to verify in Table 4 that we recorded 15 T-*patterns* of the GK's defensive actions. The T-*patterns* which occurred more frequently were related to goal defense (*n* = 16), goal exit (*n* = 14), and set pieces (*n* = 13). The recorded T-*patterns* are related to actions of goal defense, set pieces, crosses, and goal exit, and occurred in a large area. Considering the intervention forms, we can verify that technical actions that characterize the T-*patterns* are the superior deviations (set pieces and goal defense), high reception (set pieces and crossing), enchase (crossing), high lateral drop deviation (goal defense), frontal attack, 1×1 shot, and 1×1 divided (goal exit).

**Figure 3:**
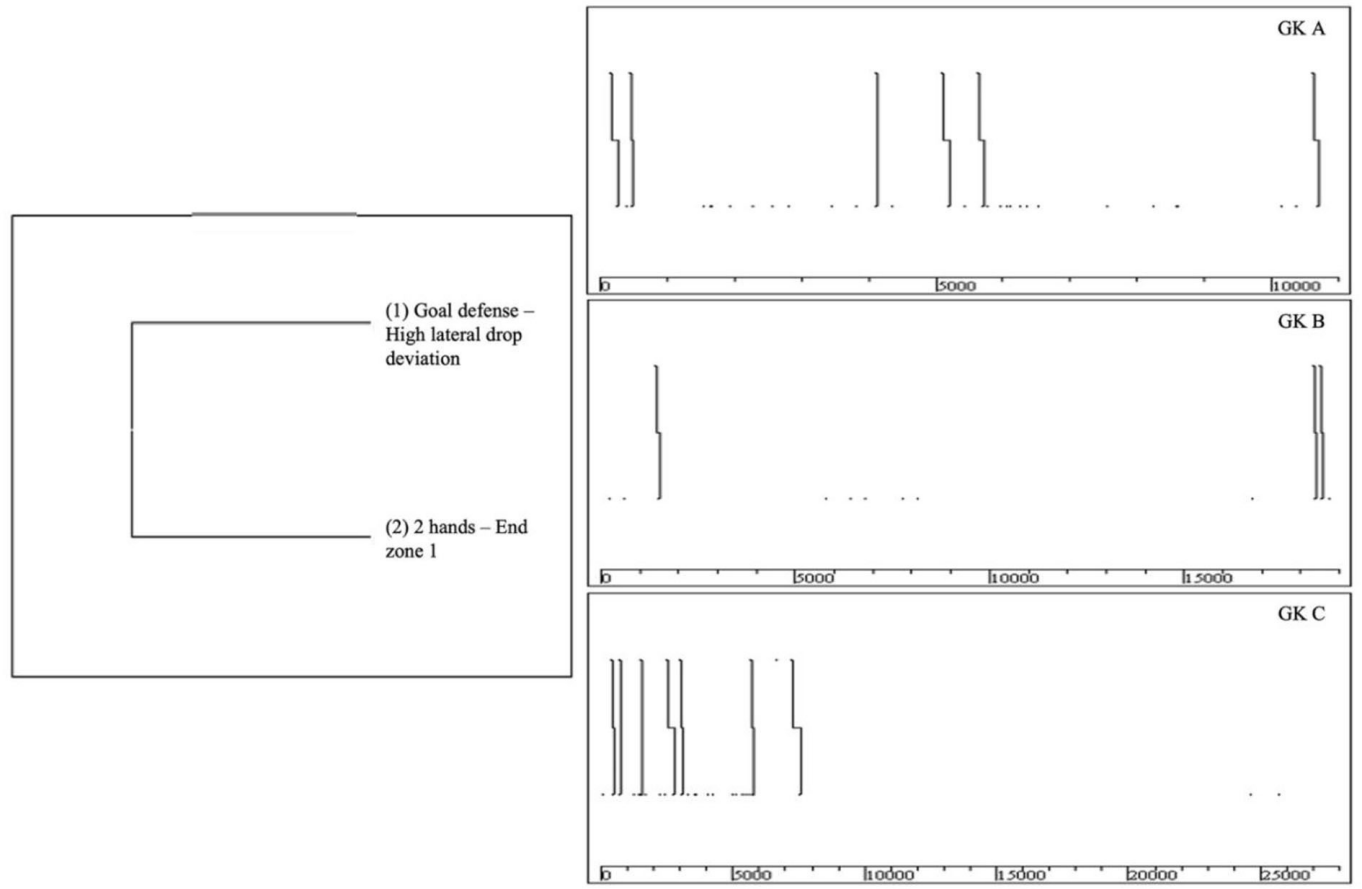
T-*patterns* of defensive actions.

Figure 3 presents the T-*patterns* of defensive action showing more occurrences of goal defense.

In Table 5, we observe T-*patterns* in which the offensive play of the GK is a result of the rules, delay of teammates, and opponent's actions, through the feet game (short and long pass) and ball replacement with the hand, contributing to the positional attack construction. The T-*patterns* with the highest occurrences are related to the circumstances the ball reaches the GK through the rules (*n* = 14 and *n* = 11) and the opponent's action (*n* = 7).

**Figure 4:**
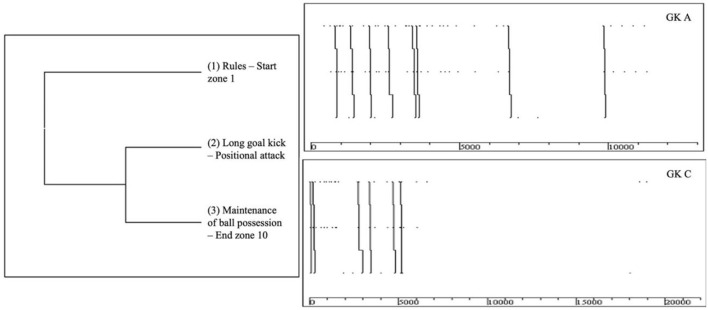
T-*patterns* of offensive actions.

Figure 4 presents the T-*patterns* of offensive actions with more occurrences.

## Discussion

The purpose of our research was to record T-*patterns* of defensive and offensive actions in official football matches in young GK. The action of GK in official matches is still a topic that needs further investigation, particularly in youth football (West, 2018; White et al., 2018). In our study, it was possible to verify the T-*patterns* of GK's defensive and offensive actions. In defensive action, we observed T-*patterns* associated with goal defense, goal exit, crosses, and set pieces. The recorded offensive T-*patterns* emphasize the importance of the game with GK's feet in today's football, and their participation in the construction of the team's offensive process is also evident (positional attack).

In the first hypothesis defined, it was proven that the T-*patterns* with more occurrences are related to the goal defense within the penalty area. In the defensive process, studies have verified that the predominant form of intervention is goal defense (Sainz de Baranda et al., 2008; Liu et al., 2015), a fact that was also evident in our study in the recorded T-*patterns*, since it demonstrates that this intervention form is associated with high lateral drop deviation, with one and two hands, in the central zones of the penalty area. Another aspect that is evident in the patterns found is that the GK's defensive actions predominantly occur within the penalty area (Sainz de Baranda et al., 2008; Lapresa Ajamil et al., 2018; Szwarc et al., 2019).

The action of GKs in set pieces also provides relevance of evidence (Santos et al., 2022), an important moment that contributes in many matches to the definition of the results (Kubayi and Toriola, 2019; Leite, 2020). T-*patterns* have been recorded in this form of intervention through the technical actions of high deviation and reception of the ball with two hands. It should be noted that there has been frequent recording of technical actions of high deviation and high reception in senior (López-Gajardo et al., 2020) and young GKs (Santos et al., 2021c, 2022).

It was also possible to verify T-*patterns* related to the form of crossing intervention, with technical action of fitting and high reception. Airspace control is of great importance to GKs, and considerable ineffectiveness in this type of action is verified in young GKs (Lapresa Ajamil et al., 2018). Developed studies show us the importance of these actions, considering the significant number of occurrences (Soares et al., 2018; Santos et al., 2021c, 2022).

In defensive terms, it was still possible to find T-*patterns* related to the goal exit (Berto and Magalhães, 2017; Lapresa Ajamil et al., 2018), with the technical execution of 1×1 shot, 1×1 frontal attack, and 1×1 divided. The literature points out that these types of actions of the GKs are more evident in high-level teams (Szwarc et al., 2010; Filho et al., 2018), since the last defensive line plays very high, and the action of the GK is fundamental for depth control.

Regarding the second hypothesis established for the study, it was partially proven. We checked T-*pattern* actions in which GK's foot play is critical, through the execution of goal kicks, short passes, and long passes. However, the final zone of the T-*patterns* of the offensive actions are the three corridors (lateral and central), a fact that is not entirely in accordance with the defined hypothesis. T-*patterns* recorded in the offensive actions showed that GK had strong participation in the construction of the offensive process of the team, through long and short passes, demonstrating the high importance of the pass technique domain (West, 2018), pass effectiveness, and corresponding maintenance of ball possession (Szwarc et al., 2010; Seaton and Campos, 2011), and that this is more evident in high-level teams (Sainz de Baranda et al., 2008; Liu et al., 2015; Lapresa Ajamil et al., 2018; Soares et al., 2018; Serrano et al., 2019; Kubayi, 2020). Another example of this was the record in T-*patterns*, where it was shaped that the ball reaches the GK through ball delays performed by teammates, reflecting the importance of GK in building the team's offensive process, as well as in varying the game center through GK to less pressure zones after retrieval of the ball (Barreira et al., 2014; López-Gajardo et al., 2020). In the moments when the ball reaches the GK through the opponent's action, it was verified in our study that the option of the GK is to perform the replacement of the ball with the hand, kick, or short passes. In situations of play in which the exit of the ball happened by the final line (rules) and the GK executes the goal kick, it was evident in the T-*patterns* the option of performing long passes to the areas of the offensive midfield (10–12).

Our research is another contribution, through the identification of T-*patterns*, aiming that young GK coaches, who also harbor a specific role in the football coaching staff, may have more information about how a GK operates in the offensive and defensive process, thus adding knowledge to a more effective training planning program. It is true that our investigation has some limitations that come into not having considered the level of the opponent, the home-away condition, as well as the outcome of the game. The number of GKs observed can also be considered a limitation of the study, a fact that should be considered when analyzing the results obtained. Future research should take these considerations into account, as well as carry out this line of research in different age groups and women's football teams.

## Practical implications

The present study contributes, through the analysis of T-*patterns*, to the increase of knowledge about the fundamental defensive and offensive technical actions performed by young football GKs, which can represent an important contribution to the planning of training by specific coaches. In defensive terms, the training of technical actions of goal defense and set pieces are fundamental to the performance of the GK action in the competition environment. For teams with very advanced defensive lines, the 1×1 confrontation actions can be decisive for the match result. With respect to offensive actions, the technical training of short and long passes should be considered important in training, considering the importance of GK actions for building the team's offensive game. It is also worth noting that the ball replacement with the hand, as a technical action, needs to be taken into consideration by the specific GK coaches.

## Conclusion

The U-17 GKs present as fundamental forms of intervention for the goal defense, goal exit, and set pieces. The execution forms evidenced in the patterns were high lateral drop deviations, high deviation, high reception, fitting, 1×1 divided, 1×1 shot, and 1×1 frontal attack. The intervention zones are predominantly those corresponding to the penalty area.

The recorded T-*patterns* demonstrate the importance of GK actions in the offensive process of the team, evidencing the need for effective execution of short and long passes. The replacement of the ball with the hand, the goal kick, and the GK kick are also techniques evidenced in the registered standards.

## Data availability statement

The raw data supporting the conclusions of this article will be made available by the authors, without undue reservation.

## Ethics statement

The studies involving human participants were reviewed and approved by Ethics Committee of the Polytechnic Institute of Leiria (CE/ IPLEIRIA/22/2021). Written informed consent to participate in this study was provided by the participants' legal guardian/next of kin.

## Author contributions

FS and JS conceived and developed the research, as well as excreted this article. ME and CF contributed to the literature review and article review. VP and PS reviewed the article and made important contributions to the various sections of the article. All authors read and approved the final version of the manuscript.

## Funding

This work was supported by national funds through FCT-Fundação para a Ciência e a Tecnologia, I.P., within the framework of the project UIDB/04748/2020.

## Conflict of interest

The authors declare that the research was conducted in the absence of any commercial or financial relationships that could be construed as a potential conflict of interest.

## Publisher's note

All claims expressed in this article are solely those of the authors and do not necessarily represent those of their affiliated organizations, or those of the publisher, the editors and the reviewers. Any product that may be evaluated in this article, or claim that may be made by its manufacturer, is not guaranteed or endorsed by the publisher.
